# Phenotypic and functional analyses show stem cell-derived hepatocyte-like cells better mimic fetal rather than adult hepatocytes

**DOI:** 10.1016/j.jhep.2014.10.016

**Published:** 2015-03

**Authors:** Melissa Baxter, Sarah Withey, Sean Harrison, Charis-Patricia Segeritz, Fang Zhang, Rebecca Atkinson-Dell, Cliff Rowe, Dave T. Gerrard, Rowena Sison-Young, Roz Jenkins, Joanne Henry, Andrew A. Berry, Lisa Mohamet, Marie Best, Stephen W. Fenwick, Hassan Malik, Neil R. Kitteringham, Chris E. Goldring, Karen Piper Hanley, Ludovic Vallier, Neil A. Hanley

**Affiliations:** 1 Centre for Endocrinology & Diabetes, Institute of Human Development, Faculty of Medical & Human Sciences, University of Manchester, Manchester Academic Health Science Centre, AV Hill Building, Oxford Road, Manchester, UK; 2 Wellcome Trust-Medical Research Council Stem Cell Institute, Anne McLaren Institute for Regenerative Medicine, Department of Surgery, Robinson Way, Cambridge CB2 0SZ, UK; 3 Wellcome Trust Sanger Institute, Hinxton CB10 1SA, UK; 4 Department of Pharmacology & Therapeutics and MRC Centre for Drug Safety Science, University of Liverpool, Sherrington Building, Ashton Street, Liverpool, UK; 5 Bioinformatics, Faculty of Life Sciences, Michael Smith Building, Oxford Road, Manchester, UK; 6 Stem Cell Research Group, Faculty of Medical & Human Sciences, University of Manchester, Manchester Academic Health Science Centre, AV Hill Building, Oxford Road, Manchester, UK; 7 Human Genetics Division, University of Southampton, Southampton General Hospital, Tremona Road, Southampton, UK; 8 North Western Hepatobiliary Unit, Aintree University Hospital NHS Foundation Trust, Longmoor Lane, Liverpool L9 7AL, UK; 9 Endocrinology Department, Central Manchester University Hospitals NHS Foundation Trust, Grafton St, Manchester, UK

**Keywords:** HLC, hepatocyte-like cell, PSC, pluripotent stem cell, ESC, embryonic stem cell, Wnt, wingless-related integration site, DE, definitive endoderm, FGF, fibroblast growth factor, BMP, bone morphogenetic protein, HGF, hepatocyte growth factor, DEX, dexamethasone, OSM, oncostatin M, AFP, alpha-fetoprotein, CYP, cytochrome P450, MEF, mouse embryonic fibroblast, IPSC, induced pluripotent stem cell, iTRAQ, isobaric tagging for relative and absolute quantification, GSTp, glutathione S-transferase π, Human embryonic stem cell, Embryo, Hepatic, Liver, Hepatotoxicity

## Abstract

**Background & Aims:**

Hepatocyte-like cells (HLCs), differentiated from pluripotent stem cells by the use of soluble factors, can model human liver function and toxicity. However, at present HLC maturity and whether any deficit represents a true fetal state or aberrant differentiation is unclear and compounded by comparison to potentially deteriorated adult hepatocytes. Therefore, we generated HLCs from multiple lineages, using two different protocols, for direct comparison with fresh fetal and adult hepatocytes.

**Methods:**

Protocols were developed for robust differentiation. Multiple transcript, protein and functional analyses compared HLCs to fresh human fetal and adult hepatocytes.

**Results:**

HLCs were comparable to those of other laboratories by multiple parameters. Transcriptional changes during differentiation mimicked human embryogenesis and showed more similarity to pericentral than periportal hepatocytes. Unbiased proteomics demonstrated greater proximity to liver than 30 other human organs or tissues. However, by comparison to fresh material, HLC maturity was proven by transcript, protein and function to be fetal-like and short of the adult phenotype. The expression of 81% phase 1 enzymes in HLCs was significantly upregulated and half were statistically not different from fetal hepatocytes. HLCs secreted albumin and metabolized testosterone (CYP3A) and dextrorphan (CYP2D6) like fetal hepatocytes. In seven bespoke tests, devised by principal components analysis to distinguish fetal from adult hepatocytes, HLCs from two different source laboratories consistently demonstrated fetal characteristics.

**Conclusions:**

HLCs from different sources are broadly comparable with unbiased proteomic evidence for faithful differentiation down the liver lineage. This current phenotype mimics human fetal rather than adult hepatocytes.

## Graphical abstract

**Figure:**
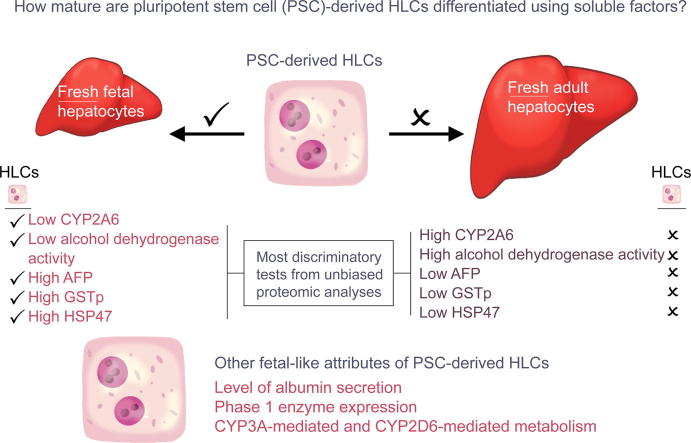

## Introduction

Hepatocyte-like cells (HLCs), differentiated from pluripotent stem cells (PSCs), offer promise as *in vitro* models of human liver development, function, and toxicity [1], [2]. Most protocols have attempted mimicry of embryogenesis through the addition of soluble factors to the media. Activin A [3], [4], [5], [6], [7], [8], [9], [10], [11], [12], [13], alone or together with Wingless-related integration site (Wnt) 3A [7], [8], [9], [14], [15], promotes definitive endoderm (DE)-like differentiation. Fibroblast growth factor (FGF) and bone morphogenetic protein (BMP) family members encourage hepatic differentiation [5], [6], [8], [9], [11], [12], [13]; and hepatocyte growth factor (HGF), the synthetic glucocorticoid, dexamethasone (DEX), and oncostatin M (OSM) support increased maturity [3], [4], [6], [8], [9], [10], [11], [12], [13], [14], [16]. However, fully mature hepatocytes have not been produced, which raises two unanswered questions: are cells aberrant because human liver development has not been followed with adequate specificity; or, if the lineage is correct, are HLCs actually ‘stuck’ in a fetal-like state? Assessment of the latter is problematic for two largely unaddressed reasons. HLC maturity is over-estimated if compared to sub-optimal adult hepatocytes. Thawed cells taken into culture are challenging to maintain [17]; in a well-controlled example, over 90% of the cytochrome P450 (CYP) 3A activity was lost in cryopreserved cells compared to freshly plated cells [18]. This illustrates the risk of over-interpreting the HLC phenotype if compared against dedifferentiated controls. Secondly, fresh fetal hepatocyte controls have been lacking when assessing HLC function. This risks misunderstanding as we have recently shown human fetal hepatocytes possess proteins, such as CYP3A4, commonly interpreted as adult markers [19].

To address these persisting questions about the differentiation and maturity of HLCs, we implemented a protocol with sufficient commonality to allow comparison with multiple previous reports. We analysed a wide range of human ESC lines, derived under different conditions alongside H9 cells, the most popular line for generating HLCs [3], [7], [9], [10], [12], [14], [18], [20]. HLCs were assessed by proteome analysis and in a series of assays against fresh human fetal and adult hepatocytes. We also included cells differentiated by a second protocol in an extended array of new tests for differentiation status, devised by unbiased proteomics and principal components analysis that distinguish fetal from fresh adult and dedifferentiated adult hepatocyte phenotypes [19].

## Materials and methods

### Human tissue and cells, and their culture

**Fig. 1:**
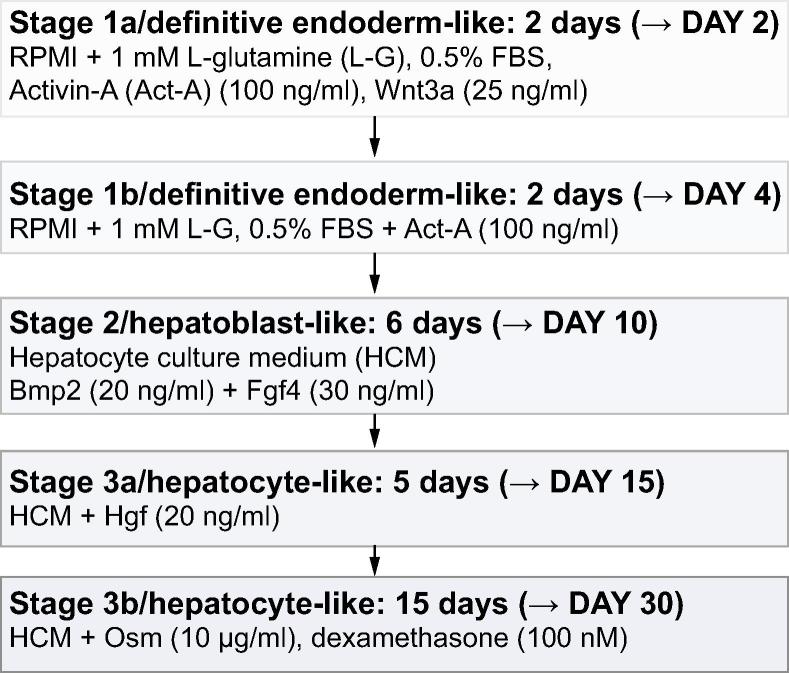
**The three-stage differentiation protocol.** RPMI, Roswell Park Memorial Institute; FBS, fetal bovine serum.

Human embryonic stem cell (ESC) lines were obtained with consent either directly from the derivation laboratory or the UK Stem Cell Bank. Cells were maintained on inactivated mouse embryonic fibroblast (MEF) cells [21]. The differentiation protocol (Fig. 1) was commenced 3–4 days post passage onto fresh MEFs using Wnt3a (R&D Systems, UK) and Activin A (Peprotech, UK), diluted in RPMI media (Sigma-Aldrich, UK); followed by BMP2, OSM, FGF2, HGF (all R&D Systems) and DEX (Sigma-Aldrich, UK), diluted in Hepatocyte Culture Medium (HCM) (Lonza, UK). Information on the human fetal and adult hepatocyte controls can be found in the Supplementary Materials and methods. Human induced pluripotent stem cells (IPSCs) were developed and differentiated as previously reported [6], [22].

### Immunoblotting, immunofluorescence, cell sorting and cell proliferation and apoptosis studies

Immunoblotting and immunofluorescence were conducted as previously reported (Supplementary Table 1) [19], [23]. Fluorescent activated cell sorting (FACS), cell proliferation and apoptosis are described in Supplementary Materials and methods.

### Protein isolation and proteomic analysis

Protein isolation from whole cell extracts and labelling for isobaric tagging for relative and absolute quantification (iTRAQ) proteomics was described by Rowe *et al.* [19]. Quantitation of proteins was relative to a common reference preparation included in each run across different experiments. Protein identification and interrogation are described in Supplementary Materials and methods.

### Phenotypic analysis

Gene expression analysis by RNA sequencing (RNA-seq) and quantitative PCR is described in Supplementary Materials and methods. Albumin and urea secretion into the media was measured using a human albumin ELISA kit and the QuantiChrom™ urea assay kit (both from Bethyl Laboratories). Comparisons with fetal and adult hepatocyte data used the unpaired two-tailed Student’s *t* test. CYP3A activity was assessed in duplicate by incubation with P450-Glo™ CYP3A4 assay reagent (Luciferin-PFBE; Promega Ltd). For CYP analysis by mass spectrometry, cells were incubated with 1 mM testosterone or 1 mM dextromethorphan (Sigma, UK) in HCM. Conditioned medium was collected and diluted 1:1 in 0.5 μM phenacetin (Sigma) stop solution in methanol. CYP activity was calculated per min incubation. Alcohol dehydrogenase activity of cell lysates was assessed using a detection kit following the manufacturer’s instructions (Abcam, UK). Results were standardized to the amount of protein measured by Bradford assay.

## Results

### Differentiation of human ESCs to HLCs

**Fig. 2:**
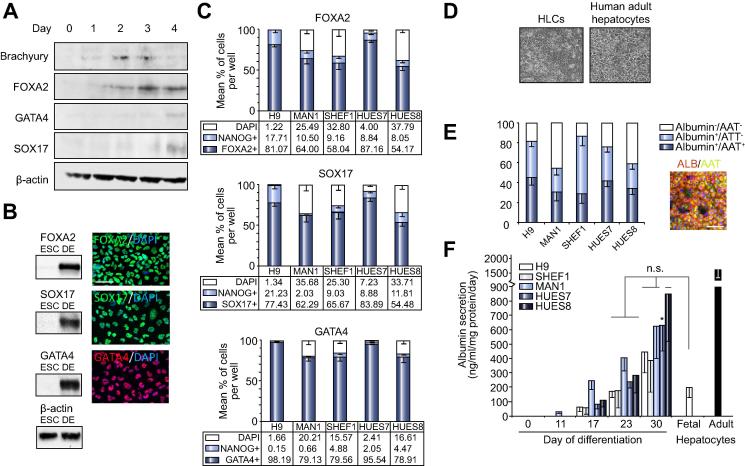
**Differentiation of ESCs to hepatocyte-like cells.** (A) Immunoblotting of ESCs differentiated towards definitive endoderm (DE). (B) Inclusion of Wnt3A for stage 1a improved detection of FOXA2, SOX17, and GATA4 by immunoblotting and immunofluorescence at day 4 (DE). Size bar = 25 μm. (C). Mean percentage of cells by count (± S.E.; numbers represented by the bar are shown below) that contained nuclear FOXA2, SOX17 or GATA4 either alone or in combination with NANOG at the end of stage 1. (D) Brightfield image of HLCs (H9 shown), compared to freshly plated human adult hepatocytes. Size bar = 150 μm. (E) Quantification by cell counting (mean ± S.E.) of dual immunofluorescence for albumin and α1-antitrypsin (AAT) in the five human ESC lines. Example image is for the H9 lineage counterstained with DAPI. Size bar = 50 μm. (F) Albumin secretion into the media (mean ± S.E. from >3 experiments) during differentiation compared to the secretion from equivalent numbers of freshly plated human fetal and adult hepatocytes. n.s., no significant difference between day 23 and day 30 for all lines (except HUES7 at day 30, ^∗^*p* <0.05) and fetal hepatocytes. Values for all lines except HUES8 (n.s.) were significantly lower (*p* <0.05) than for adult hepatocytes.

Based on previous studies [3], [4], [5], [6], [7], [8], [9], [10], [11], [12], [13], [14], [15], [16], iteration of a 3-stage protocol (Fig. 1) was devised to differentiate a range of human ESC lines, derived under diverse conditions to HLCs. During stage 1, Brachyury protein was increased by Activin A on day 2–3, at and after which FOXA2, GATA4 and SOX17 increased (Fig. 2A). However, the low serum caused significant cell death, which was prevented by Wnt3A (25 ng/ml) for the first two days of culture [7], [8], [9], [14], [15], leading to robust detection of the three nuclear transcription factors by day 4 (Fig. 2B). FOXA2, SOX17, and GATA4 were detected in >50% of cells for each ESC line, indicating a shared but variable propensity for DE-like differentiation. More homogeneous differentiation was observed in H9 cells (77–98% of cells positive for FOXA2, SOX17, and GATA4) and HUES7 cells (84–96% cells positive for the three transcription factors) (Fig. 2C and Supplementary Fig. 1A). At the end of stage 2 (hepatoblast-like cells), 91% of HUES7 and 98% of H9 derivatives contained AFP, of which at least two-thirds clearly dual-stained for nuclear HNF4α (Supplementary Fig. 1B and C; only robust HNF4α staining was counted most likely underestimating the entire population of HNF4α^+^/AFP^+^ cells). Approximately 25% of these AFP^+^ cells were proliferating according to BrdU uptake over 4 h, with 10–15% in apoptosis as detected by caspase-3 activity for both HUES7 and H9 derivatives (Supplementary Fig. 1D).

### Initial characterization of HLCs

Transcripts for albumin and alpha1-antitrypsin (*AAT*, officially designated *SERPINA1*) were barely identified in DE-like cells but were readily detected in early HLCs following stage 3A (Supplementary Fig. 2A and B). We have previously shown that the transcription factors GATA4 and SOX17 become restricted from the early human embryonic liver compared to the adjacent foregut [23]. Between DE-like cells and early HLCs, *GATA4*, and *SOX17* expression declined by approximately 75% and >90% respectively (data not shown). The transcription factor HNF4α encoded by *HNF4A*, is a master regulator of the hepatocyte phenotype [24], [25], [26]. During development there is a switch from an upstream immature P2 promoter to a downstream ‘liver’ P1 promoter, generating alternative first exons of the *HNF4A* gene [27], [28]. This was mirrored in our cultures: in DE-like cells, *HNF4A* was expressed from the ‘immature’ P2 promoter; however, in HLCs the downstream first exon was preferentially transcribed from the P1 ‘liver’ promoter (Supplementary Fig. 2C, red boxes).

Final HLC morphology mimicked that of freshly plated human adult hepatocytes (Fig. 2D). Across the five different ESC lines, albumin was present in >75% of differentiated H9, SHEF1 and HUES7 cells (Fig. 2E). At least half of the H9 and HUES7 albumin-positive HLCs also contained AAT (Fig. 2E). All five cell lines showed a progressive increase in albumin secretion starting from day 11 (Fig. 2F). At the end of differentiation, levels were at least comparable to those from freshly plated fetal hepatocytes, which were approximately 8-fold lower than those from freshly plated adult hepatocytes. HLCs also showed urea secretion (mean ± S.E.: 2.87 ± 0.18 μg/ml/mg protein/day) comparable to fresh fetal hepatocytes (2.71 ± 0.09 μg/ml/mg protein/day), but approximately 18-fold lower than freshly plated adult cells (50.6 ± 6.11 μg/ml/mg protein/day).

We wanted to assess whether HLCs mimic periportal or pericentral hepatocytes. Glutamine synthase, a pericentral marker, was readily detected by immunocytochemistry in contrast to carbamoyl-phosphate synthase, a periportal protein (Supplementary Fig. 3A). This correlated to their transcript profiles (Supplementary Fig. 3B) and was true for a range of other genes, differentially expressed between pericentral and periportal hepatocytes [29]. All the pericentral genes except UDP-glucuronosyltransferase 1A (*UGT1A*) were expressed at increased or equivalent levels to those in DE-like cells. In contrast, only phosphoenolpyruvate carboxykinase 2 (*PCK2*) of the periportal markers was expressed in HLCs, but at levels lower than in DE-like cells.

**Fig. 3:**
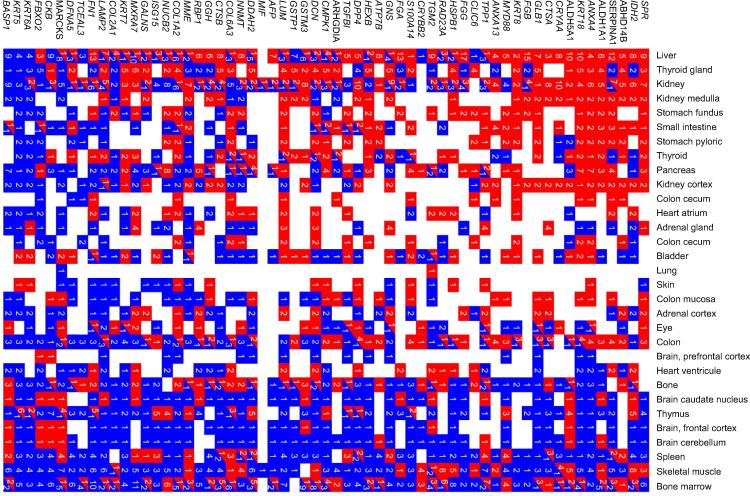
**Proteins upregulated in HLCs characterize liver more than other human organs and tissues.** Heatmap of the 61 proteins significantly upregulated (>2-fold) in H9 HLCs compared to undifferentiated ESCs (Supplementary Table 2) analysed against gene expression experiments from a wide range of human organs and tissues, deposited in the EMBL/EBI Gene Expression Atlas (GEA). The numbers in individual red or blue boxes represent the number of experiments deposited in the GEA database where the gene, encoding that particular gene was up- (red) or downregulated (blue) in the relevant organ or tissue type.

While these data were encouraging of liver-specific differentiation, it is an assumption based on limited user-selected proteins and assays and ignores potential similarity to cell types from other organs. We performed unbiased proteomic assessment of whole cell extracts from undifferentiated H9 cells and their HLCs at the end of stage 3. 61 proteins showed significant upregulation (>2-fold) including known liver markers, such as AAT/SERPINA1 and phase 1 (e.g. aldehyde dehydrogenases) and phase 2 enzymes (e.g., nicotinamide N-methyltransferase [NNMT] and glutathione S-transferase [GST] Mu3) (Supplementary Table 2). Cytokeratin (KRT) 8 and KRT18, both upregulated, function together as heteropolymers to protect hepatocytes from mechanical and non-mechanical stress [30]. KRT7, apparent in <10% of cells by immunocytochemistry (data not shown), may reflect slight permissiveness in our protocol to cholangiocyte differentiation but has also been reported as a hepatocyte progenitor cell marker [31], [32]. Some upregulated proteins were not recognized as hepatocyte markers, such as KRT5 and KRT6A. Therefore to assess the broad proteome phenotype, we compared the upregulated protein dataset against data from 30 other human organs and tissues in the EBI Gene Expression Atlas (Fig. 3). By heatmap, the upregulated HLC proteome most closely resembled the liver, followed by another anterior derivative of foregut endoderm, the thyroid. Other foregut endoderm derivatives (stomach, small intestine and pancreas) showed recognizable similarity, in contrast to a marked divergence of HLCs from mesodermal and ectodermal derivatives such as bone marrow, skeletal muscle and brain.

### HLCs have a metabolic profile comparable to human fetal rather than adult hepatocytes

**Fig. 4:**
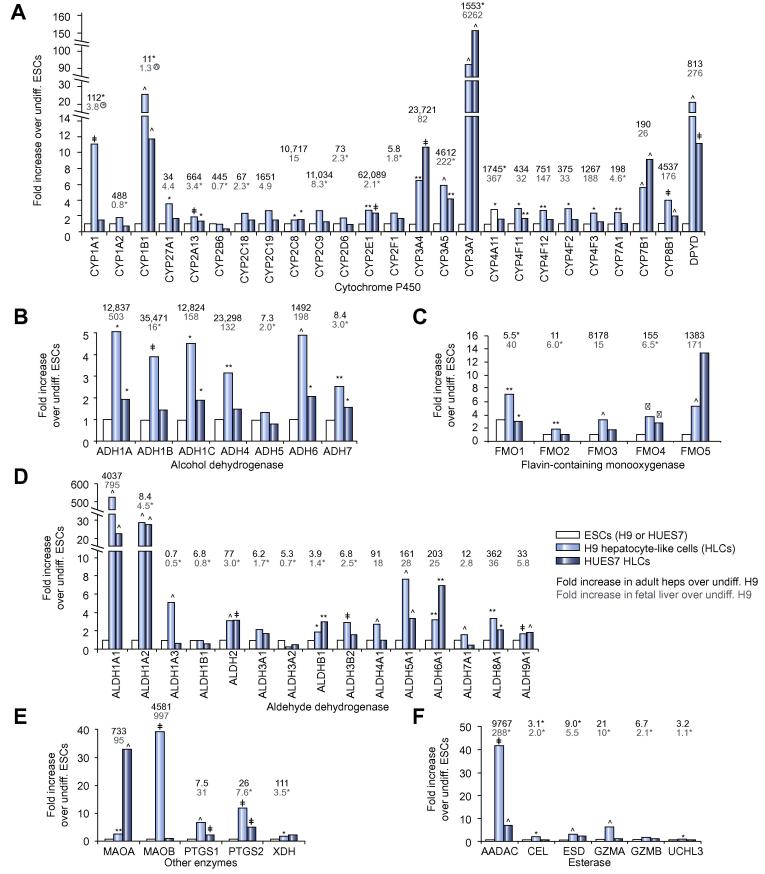
**Quantitative RT-PCR expression analysis of genes encoding phase 1 enzymes.** (A–F) Genes encoding different classes of phase 1 enzymes. Gene expression in HLCs, quantified as fold difference over levels in the corresponding undifferentiated ESCs. Fold difference in fresh human adult hepatocytes (black numbers above the bars) and fresh human fetal hepatocytes (grey numbers above the bars) are relative to levels in undifferentiated H9 cells. For light blue (H9 HLCs) and dark blue (HUES7 HLCs) symbols next to bars: ^∗^*p* <0.05, ^∗∗^*p* <0.02, ^†^*p* <0.01, ^‡^*p* <0.005, ^^^*p* <0.001 compared to their parent undifferentiated ESCs. For symbols next to the black (adult) or grey (fetal) numbers, ^∗^ indicates no statistical difference from H9 HLCs; when encircled (for *CYP1A1* and *CYP1B1*) the H9 HLC level was statistically higher than fetal or adult expression levels.

To gain broad developmental insights iTRAQ was performed on whole cell extracts rather than enriched microsomes. Consequently, this restricted identification of phase 1 enzymes, especially CYPs, similar to our previous study of human fetal and adult hepatocytes [19]. We used quantitative RT-PCR to analyse the expression of phase 1 enzymes, including CYPs (Fig. 4A), alcohol dehydrogenases (Fig. 4B), flavin-containing monooxygenases (Fig. 4C), aldehyde dehydrogenases (Fig. 4D), esterases (Fig. 4F), and other enzymes (Fig. 4E). The expression of 51/63 enzymes (81%) was significantly increased in H9 HLCs and 30 (48%) enzymes were significantly increased in HUES7 HLCs compared to their undifferentiated counterparts. *CYP3A* family members, *CYP1B1*, and *DPYD* were clearly increased in HLCs (Fig. 4A) with more modest increases in a number of alcohol dehydrogenases and flavin-containing monooxygenases (Fig. 4B and C). The increase in the aldehyde dehydrogenase gene, *ALDH1A1*, in H9 HLCs approached levels in fetal hepatocytes, while *ALDH1A2* levels surpassed those of both adult and fetal cells (Fig. 4D). However, more generally, HLC transcript levels were markedly reduced compared to those in fresh human adult hepatocytes. In contrast, expression of 33 of the 63 phase 1 enzymes in H9 HLCs (52%) was statistically either greater or no different than in fresh human fetal hepatocytes, indicating a major overlap between the HLC and fetal phase 1 metabolic phenotype.

**Fig. 5:**
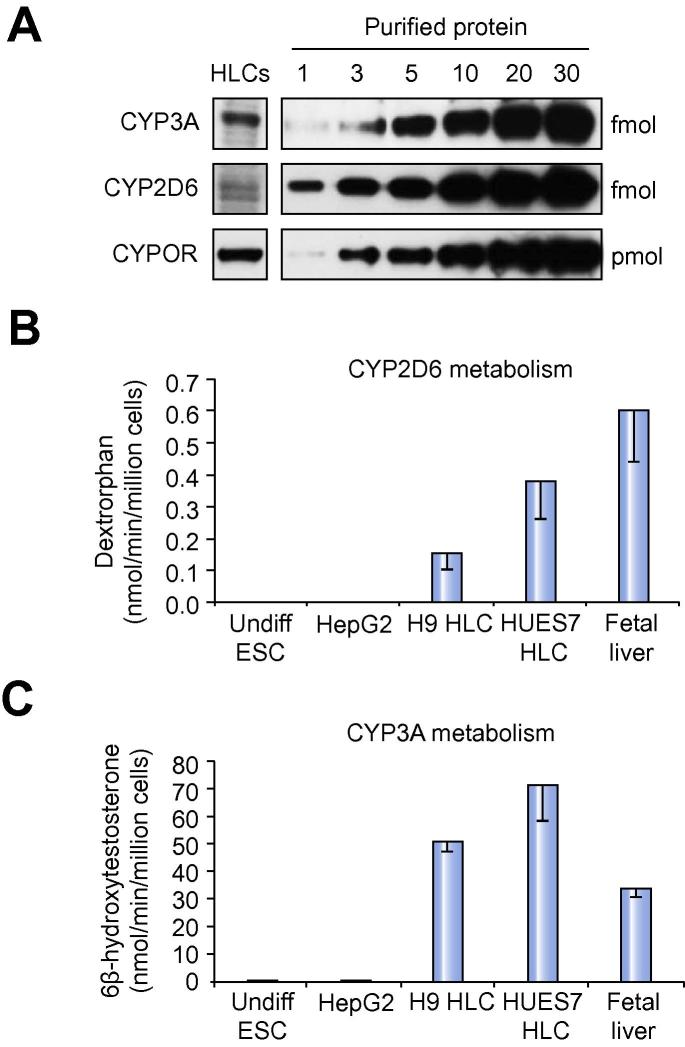
**HLCs are metabolically similar to fetal hepatocytes.** (A) Immunoblotting for CYP3A, CYP2D6 and CYPOR in HLCs. Equivalent numbers of human adult hepatocytes contained approximately 30 fmol of CYP3A and 20 fmol of CYP2D6. (B) CYP2D6 metabolism of dextromethorphan to dextrorphan. Conversion in fresh adult hepatocytes was 200 nmol/min/million cells. (C) CYP3A metabolism of testosterone to 6β-hydroxytestosterone. Conversion in fresh adult hepatocytes was 3300 nmol/min/million cells. Bar graphs in (B-C) show mean ± S.E. from >3 independent experiments.

CYP3A activity, mostly via CYP3A4 in adult liver, and CYP2D6 are two major mechanisms for drug metabolism that require CYP oxidoreductase (CYPOR) to donate electrons during catalysis. To help further gauge maturity of HLCs, we measured protein levels by immunoblotting and metabolic activity by mass spectrometry. Immunoreactivity for CYP3A (current antibodies fail to distinguish the different CYP3A isoforms) in HLCs was approximately 10% of the levels in fresh adult hepatocytes (Fig. 5A). For CYP2D6, two closely positioned bands were visible, the upper of which corresponded to the size of the purified protein (Fig. 5A). CYPOR was robustly detected. CYP2D6 metabolizes dextromethorphan to dextrorphan. This activity was detected in both H9 and HUES7 HLCs, but not in ESCs or HepG2 cells. Conversion by HUES7 HLCs was similar to that by fetal hepatocytes but was 527-fold less than detected in fresh adult hepatocytes (1313-fold lower than adult cells for H9 HLCs) (Fig. 5B). CYP3A-mediated metabolism of testosterone to 6β-hydroxytestosterone by HLCs was at least 100-fold greater than by ESCs or HepG2 cells and at least comparable to fresh fetal hepatocytes (Fig. 5C). Nevertheless, CYP3A metabolism of testosterone was 47-fold and 66-fold higher in fresh adult hepatocytes than in HUES7 and H9 HLCs, respectively. Mass spectrometry provides a ‘gold standard’ for metabolic assay. In contrast, our experience of measuring CYP3A4 activity by commercially available luciferase assay (PFBE reagent, Promega) was unhelpful. Although HLCs from all five ESC lines matched fresh fetal hepatocytes, fetal hepatocytes misleadingly showed greater activity than their adult counterparts (Supplementary Fig. 4).

### Bespoke tests to distinguish hepatocyte maturity show that HLCs are fetal-like

**Fig. 6:**
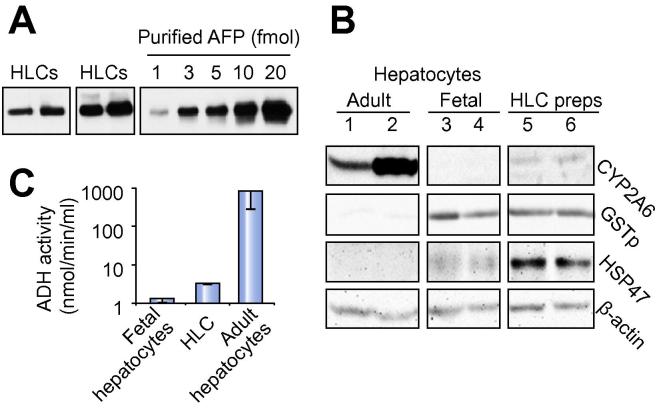
**Bespoke tests to distinguish hepatocyte maturity.** (A–C) Tests determined by principal components analysis to discriminate human fetal and adult hepatocytes [19]. (A) Immunoblotting of four different HLCs differentiations for the fetal marker AFP. (B) Immunoblotting of fresh adult (lanes 1 and 2) and fetal hepatocytes (lanes 3 and 4), and two different HLC preparations (lanes 5 and 6) for the most discriminatory adult hepatocyte marker CYP2A6 and fetal markers, GSTp and HSP47 [19] with β-actin as control. (C) Assay for alcohol dehydrogenase activity in HLCs compared to fetal and adult hepatocytes. Mean ± S.E. from 3 experiments using different HLC preparations and different fetal and adult samples.

**Fig. 7:**
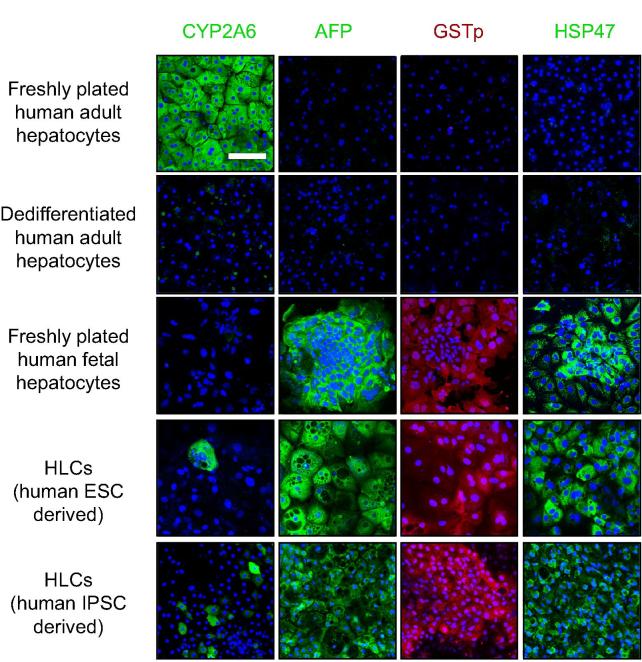
**Immunocytochemistry to discriminate hepatocyte maturity.** Immunofluorescence for CYP2A6, AFP, GSTp, and HSP47 on freshly plated adult human hepatocytes, dedifferentiated human adult hepatocytes, freshly plated human fetal hepatocytes, and HLCs derived from human ESCs and from human IPSCs via an alternative protocol [6]. Examples shown are from one set of experiments, which were performed in triplicate on three separate cell preparations/differentiations. Size bar represents 100 μm for all panels.

**Fig. 8:**
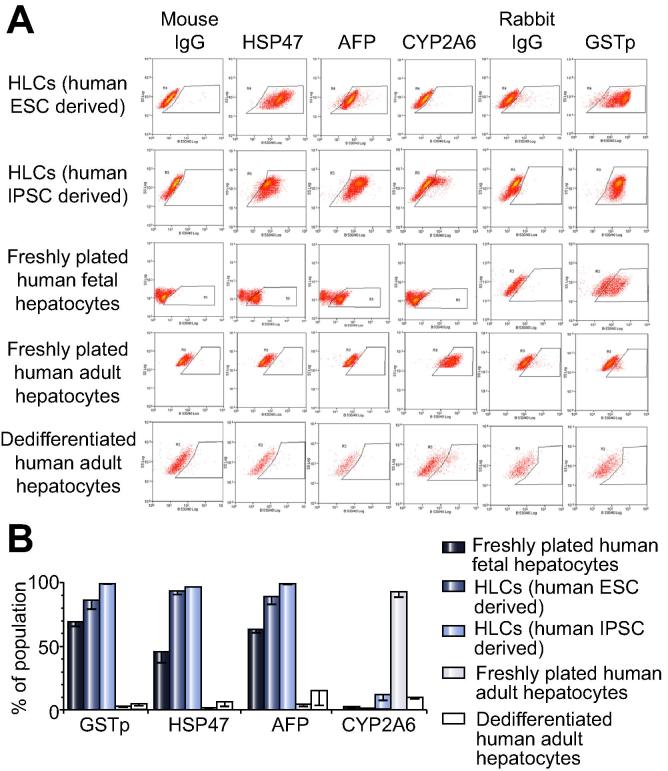
**Flow cytometry analysis to discriminate hepatocyte maturity.** (A) One example of flow cytometry experiments for CYP2A6, AFP, GSTp, and HSP47 with their corresponding immunoglobulin control on freshly plated adult human hepatocytes, dedifferentiated human adult hepatocytes, freshly plated human fetal hepatocytes, and HLCs derived from human ESCs and from human IPSCs via an alternative protocol [6]. These flow cytometry experiments were each performed on three separate preparations of each cell type. (B) Graph showing mean ± S.E. from combining the three individual flow cytometry experiments, as in (A), for each marker and cell type.

We have previously shown that CYP3A4 protein, detected by iTRAQ, is relatively ineffective at determining hepatocyte maturity or whether cells have dedifferentiated [19]. In contrast, principal components analysis provided new protein combinations and simple assays not requiring mass spectrometry to distinguish human adult hepatocytes from their fetal counterparts: AFP, GSTp and heat shock protein (HSP) 47 with negligible alcohol dehydrogenase (ADH) activity or CYP2A6 discriminates fetal cells; conversely, abundant CYP2A6 and ADH activity are hallmarks of adult cells [19]. AFP, GSTp, and HSP47 were readily detected in our HLCs (Fig. 6Aa and B). CYP2A6, another marker of perivenous cells, was weakly detected in HLCs, which also showed slightly more ADH activity than fetal cells (Fig. 6C); however, both CYP2A6 levels and ADH activity were much higher in adult hepatocytes. Although our protocol has marked similarity to that used widely by others [3], [4], [5], [6], [7], [8], [9], [10], [11], [12], [13], [14], [15], [16], we wanted to extend these discriminatory tests further by adding immunocytochemistry and FACS analysis of HLCs differentiated from IPSCs by another very well-established protocol in a different lab [6]. We also examined whether our markers could distinguish dedifferentiated adult hepatocytes from fetal-like immaturity. Although we were unable to analyse ADH by immunocytochemistry, CYP2A6, AFP, GSTp and HSP47 all gave the expected distribution in triplicated samples of freshly plated human adult and fetal hepatocytes (Fig. 7). In contrast, minimal staining was apparent for any of the markers in dedifferentiated adult hepatocytes. HLCs from both sources produced fetal-like staining patterns with few cells stained for CYP2A6. The corresponding profiles were evident by FACS (Fig. 8). Taken together, these data consistently indicate a fetal phenotype for HLCs.

## Discussion

Our differentiation of DE-like cells matched others in its sequential use of Activin A [3], [4], [5], [6], [7], [8], [9], [10], [11], [12], [13] and Wnt3A [7], [8], [9], [14], [15], FGF and BMP signalling [5], [6], [8], [9], [11], [12], [13], HGF [3], [4], [8], [9], [10], [11], [12], [13], and either OSM [6], [8], [10], [13], [16] or dexamethasone [4], [9] or both (this study and [3], [11], [14]). Our chosen stem cell lines covered a range of derivation conditions with similar results from additional SHEF and H1 lines (data not shown). H9 and HUES7 cells gave the highest percentage of conversion to a hepatocyte-like phenotype similar to that achieved by others, using H9 cells [3], [7], [9], [10], [12], [14], [18], [20].

Four features of our data extend confidence that our protocol, and by inference others [3], [4], [5], [6], [7], [8], [9], [10], [11], [12], [13], [14], [15], [16], mimic normal human liver development. First, the downregulation of *SOX17* and *GATA4* expression between DE-like cells and HLCs (this study and [7], [10], [12]) complies with the exclusion of these transcription factors, which we recently showed in human liver bud as it develops from the foregut between 4 and 5 weeks post conception [23]. The high rates of proliferation and apoptosis during this phase of differentiation match those seen previously [25] and are consistent with the major remodelling that characterizes embryogenesis. We mimicked the change in *HNF4A* promoter usage during embryonic development [27], [28]. Finally, despite some unexpected individual proteins, we demonstrated that the HLC proteome had more in common with human liver than any other organ tested. These novel unbiased protein data complement a previous ‘liver-specific’ transcriptome signature defined in HLCs [10]. The latter emanated from microarray analysis of multiple adult human tissues [33]. ‘Liver-specific’ genes, such as albumin (*ALB*), various *CYPs*, and *AFP*, can be expressed more widely than in liver [1]. Hence, there is reassurance from this first proteomic approximation of HLCs to liver and related endoderm fates but not to major ectoderm and mesoderm lineages.

Uncertainty on HLC maturity has been discussed by others [16], [20]; functional comparison with fresh fetal hepatocytes has been lacking [6]. We included undifferentiated stem cells and both fresh first trimester fetal and adult hepatocytes and consistently demonstrated major similarities with the fetal rather than the adult cell type. Our data were consistent with a previous report that included cryopreserved human fetal hepatocytes from 25 weeks of gestation [34]. Moreover, by studying fold increments over undifferentiated stem cells our data can be integrated with those from others. Our fetal-like HLC albumin secretion showed at least a 200-fold increase from the parent ESC line (Fig. 2F). This increment matches or surpasses that reported by others [3], [9], [11], [18], [22], [35], [36] but was still lower on average than secretion from freshly plated adult cells. However, lower increments in albumin secretion from ESCs to HLCs have been reported to match adult hepatocytes following cryopreservation [35], [36]. These data imply the ease with which adult control cells can dedifferentiate and that in fact HLCs, differentiated through the use of soluble factors, mimic human fetal cells in keeping with the common AFP detection [3], [5], [6], [7], [9], [10], [11], [12], [14], [16], [22], [37]. A similar fetal-like conclusion can be drawn from urea secretion. Others have observed relatively low urea secretion in HLCs compared to adult cells [9], [11], [36], which may also reflect a pericentral rather than a periportal phenotype [29].

H9 HLCs matched fetal hepatocytes for the expression of half of the CYPs tested but were inferior for others (e.g. *CYP2C8*, *CYP3A*, *CYP4*, and *CYP7* family members, and *CYP8B1*). This included transcripts for what have been previously considered ‘adult’ CYPs [6], warning that detection alone does not reliably indicate maturity. Phase 1 enzyme expression was commonly massively higher for fresh adult cells (up to 62,000-fold over ESCs for *CYP2E1*). This was not apparent in a recent protocol [38], using cryopreserved adult hepatocyte controls that had transcript levels only approximately 20-fold higher than undifferentiated stem cells for *CYP2B6* (445-fold here), 10-fold for *FMO3* (8178-fold here), and 20-fold for *CYP2A13* (664-fold here). The human fetal liver contains both CYP3A4 and CYP2D6 proteins prior to mid-gestation [19]. Here we showed fetal-like HLC function by mass spectrometry, despite HLCs possessing between 10- and 60-fold lower transcript levels than fetal hepatocytes for *CYP3A4*, *CYP3A5*, and *CYP3A7* (Fig. 6). Our experience with a commonly used commercial ‘CYP3A4’ luciferase assay was misleading. Luciferase CYP3A activity from HLCs was equivalent to data from others [14] and to fetal hepatocytes; but fetal hepatocytes showed more luciferase activity than adult hepatocytes, which is incompatible with the approximate 100-fold superiority of adult cells by mass spectrometry. Instead, we extended our previous discovery of a signature of proteins, capable of distinguishing fetal from adult hepatocytes [19]. Here, by adding immunocytochemistry and FACS, the combination of CYP2A6, AFP, GSTp and HSP47 accurately distinguished human fresh adult and fetal and dedifferentiated adult hepatocytes. HLCs, generated by our main protocol and another established one [6], possessed fetal discriminators rather than an adult or a dedifferentiated phenotype.

In summary, our HLC differentiation, and by inference that of others, mimics human liver development. However, by a wide range of analyses, these HLCs share pronounced similarities with human fetal rather than adult hepatocytes. This is important as it implies currently elusive soluble factors require discovery for the transition of HLCs *in vitro* into a truly mature adult phenotype.

## Financial support

This work was funded by the Stem Cells for Safer Medicine Consortium (grants to NAH and CEG), the 10.13039/501100000266Engineering and Physical Sciences Research Council (to NAH), and a 10.13039/501100000265Medical Research Council (MRC) Centre grant. NAH is a Wellcome Trust Senior Fellow (funded by WT088566 and WT097820). SW is a 10.13039/501100000268Biotechnology and Biological Sciences Research Council (BBSRC) PhD student.

## Conflict of interest

The authors who have taken part in this study declared that they do not have anything to disclose regarding funding or conflict of interest with respect to this manuscript.

## Authors’ contributions

MB, SW, SH, C-PS, FZ, RA-D, CR, DTG, RS-Y, RJ, JH, AAB, LM, and MB all contributed to the acquisition of data, its analysis and interpretation; SWF and HM assisted with the acquisition of fresh human adult hepatocytes; NRK, CEG, KPH, LV, and NAH all oversaw experiments. NAH was in charge of the overall conception and design of the study.
