# Supplementary material for: Phenotypic and functional analyses show stem cell-derived hepatocyte-like cells better mimic fetal rather than adult hepatocytes
Source: J Hepatol. 2015 Mar;62(3):581–9. doi: 10.1016/j.jhep.2014.10.016 (PMC4334496; doi:10.1016/j.jhep.2014.10.016)
Supplement: Supplementary data [file mmc1.pdf]

Supplementary data to:

Phenotypic and functional analyses show stem cell-derived hepatocyte-like cells better mimic fetal rather than adult hepatocytes

Melissa Baxter[1,†], Sarah Withey[1,†], Sean Harrison[1,†], Charis-Patricia Segeritz[2], Fang Zhang[3], Rebecca Atkinson-Dell[1], Cliff Rowe[1,3], Dave T. Gerrard[1,4], Rowena Sison-Young[3], Roz Jenkins[3], Joanne Henry[3], Andrew A. Berry[1], Lisa Mohamet[5], Marie Best[6], Stephen W. Fenwick[7], Hassan Malik[7], Neil R. Kitteringham[3], Chris E. Goldring[3], Karen Piper Hanley[1], Ludovic Vallier[2], Neil A. Hanley[1,8,*]

[1]Centre for Endocrinology & Diabetes, Institute of Human Development, Faculty of Medical & Human Sciences, University of Manchester, Manchester Academic Health Science Centre, AV Hill Building, Oxford Road, Manchester, UK; [2]Wellcome Trust-Medical Research Council Stem Cell Institute, Anne McLaren Institute for Regenerative Medicine, Department of Surgery, Robinson Way, Cambridge CB2 0SZ, UK; and Wellcome Trust Sanger Institute, Hinxton, CB10 1SA, UK; [3]Department of Pharmacology & Therapeutics and MRC Centre for Drug Safety Science, University of Liverpool, Sherrington Building, Ashton Street, Liverpool, UK; [4]Bioinformatics, Faculty of Life Sciences, Michael Smith Building, Oxford Road, Manchester, UK; [5]Stem Cell Research Group, Faculty of Medical & Human Sciences, University of Manchester, Manchester Academic Health Science Centre, AV Hill Building, Oxford Road, Manchester, UK; [6]Human Genetics Division, University of Southampton, Southampton General Hospital, Tremona Road, Southampton, UK; [7]North Western Hepatobiliary Unit, Aintree University Hospital NHS Foundation Trust, Longmoor Lane, Liverpool L9 7AL, United Kingdom; [8]Endocrinology Department, Central Manchester University Hospitals NHS Foundation Trust, Grafton St, Manchester, UK

Table of Contents

Supplementary Materials and methods 3
Human tissue and control cells 3
Flow cytometry analyses..3
Cell proliferation and apoptosis studies..4
RNA-sequencing and quantitative RT-PCR analysis of genes encoding phase 1 enzymes..4

Proteomic analysis 6
Supplementary Figures...7
Supplementary Tables...11
Supplementary References 14

Supplementary Materials and methods

Human tissue and control cells

Information on collecting the human fetal and adult hepatocyte controls, and sourcing and culturing HepG2 cells has been reported previously [20]. In brief, fetal material was obtained from voluntary termination of pregnancy with informed consent under ethical approval. Samples ranged from 9 to 14 weeks post-conception (wpc). In brief, fetal liver was cut into small sections and treated with Collagenase B (Roche Diagnostics Ltd 11088815; 2 mg/ml in HBSS+) for 30 min at 37°C. Dissociated cells were passed through a 70 µm filter, washed three times in PBS by centrifugation and viable cells seeded onto 6-well tissue culture plates at $10x10^6$ cells/well in Williams E medium with 5% FBS, 1% penicillin/streptomycin, 2mM L-glutamine and 1% Insulin-Transferrin-Selenium (ITS). Cells were fixed for future immunostaining or flow cytometry after 24h. Fresh human adult hepatocytes were obtained and quality assessed as reported previously [20] or following surgical resection. The latter tissue was used in the immunocytochemistry and FACS studies (Figs. 6-7). In brief, liver was perfused with Hepes buffered saline until blanched and then perfused with Hepes buffered saline containing 0.5 mg/ml collagenase and 0.7 mM/ml CaCl until visibly dissociating prior to manual dissociation and passage through a mesh (to remove large tissue pieces and debris). Cells were centrifuged and washed twice with hepatocyte culture media (Williams E medium supplemented with 2mM L-Glutamine, Insulin-Transferrin-Selenium (ITS) and 100nM dexamethasone), checked for viability with trypan blue, and seeded onto Matrigel-coated 6-well plates overlaid 3h later with a second layer of Matrigel (ECM-sandwich) and fresh media for analysis after 24h [20]. To purposefully dedifferentiate adult human hepatocytes, plated cells were maintained in extended culture for 30 days with media exchange every 2 days, following which cells were fixed either for immunocytochemistry or flow cytometry.

Flow cytometry analyses

Cells were fixed either at the end of the differentiation period (HLCs), 24h after plating (freshly plated hepatocytes) or after 30 days (dedifferentiated adult hepatocytes). In brief, TrypLE was added to cells (1ml/well of a 6-well plate) followed by incubation at 37°C until

cells began to dissociate. Cells were centrifuged and resuspended in 1% PFA for 10 min at room temperature followed by 2 min on ice. Cells were centrifuged and resuspended in ice-cold 70% methanol overnight at -20°C. Cells were washed in PBS three times and kept in PBS at 4°C. For flow cytometry, fixed cells were centrifuged and resuspended in FC buffer (PBS, 5% FBS, 0.1% TritonX). The relevant antibody (Supplementary table 1) or control IgG was added followed by incubation for 30 min on ice and washing cells twice in PBS. Secondary antibody (at 1:1000) was added to FC buffer and cells incubated for 20 min on ice. Cells were washed and flow cytometry carried out. AlexaFlour® 488 was excited using the 488nm laser and emission was detected using a 530/40 band pass filter. IgG controls were used in order to set gates for positive cells.

Cell proliferation and apoptosis studies

Bromodeoxyuridine (BrdU; 10µM; Sigma) was incubated with cells at the end of Stage 2 for 4h at 37°C. Cells were washed with phosphate buffered saline (PBS), dissociated with TrypLE Express (Invitrogen), centrifuged, resuspended in 200µl of ice-cold PBS / EDTA (5mM) and fixed in ice-cold ethanol. Nuclei were released with pepsin (0.4mg/ml in 0.1N HCl; Sigma) for 30min at room temperature followed by washing in PBS, re-suspension in 2N HCl, incubation in the dark for 30min at 37°C, prior to treatment with 0.1M sodium tetraborate. Further washing in PBS was followed by treatment for 30min with RNaseA (10µg/ml; R4875 Sigma). Nuclei were washed, resuspended and incubated in 10µl containing anti-BrdU antibody (AlexaFlour®488 conjugate, Millipore,) or IgG_{2a} control (AlexaFlour®488 conjugate, Santa Cruz) at 4°C overnight. All nuclei were stained with propidium iodide (50µg/ml; Sigma) and analyzed by flow cytometry within 48h. Apoptosis was studied in dissociated hepatoblast-like cells with the NucView 488 caspase 3 kit (Biotium Inc) according to the manufacturer's instructions prior to analysis by flow cytometry. In both cell proliferation and apoptosis studies 10,000 cells were gated with aggregates and debris excluded from analyses.

RNA-sequencing and quantitative RT-PCR analysis of genes encoding phase 1 enzymes

RNA sequencing (RNA-seq) libraries were generated using the TruSeq® Stranded mRNA assay (Illumina, Inc.) according to the manufacturer's protocol. Briefly, total RNA (1 □g) was used as input material from which polyadenylated mRNA was purified using polyT,

oligo-attached, magnetic beads. mRNA was reverse transcribed into first strand cDNA using random primers. Second strand cDNA was synthesized using DNA Polymerase I. Following a single 'A' base addition, adapters were ligated to the cDNA fragments, and the products then purified and enriched by PCR to create the final cDNA library. The loaded flow-cell was then paired-end sequenced on an Illumina HiSeq2000. Demultiplexing of the output data (allowing one mismatch) and BCL-to-Fastq conversion was performed with CASAVA 1.8.3. Paired end reads were mapped to hg19 using TopHat (version 1.4.1) (1) and visualized using the UCSC Genome Browser (http://genome.ucsc.edu/) (2). Gene-level transcription abundances (read counts) were calculated in the Partek Genomics Suite (version 6.6 (6.12.1227); Partek Inc., St. Louis, MO, USA). Following mapping, read counts were filtered for mitochondrial genes, ribosomal RNAs and two other multilocality RNAs ("Metazoa_SRP", “7SK”).

Phase 1 enzyme expression was analyzed in triplicate by reverse transcription and quantitative PCR using Human Phase 1 Enzymes PCR Array and accompanying reagents according to the manufacturer’s protocols (SABiosciences Ltd). Total RNA was extracted and genomic DNA eliminated for each sample. A reverse transcription cocktail mix was prepared as instructed and was added to a standardized amount of each RNA sample. First strand cDNA synthesis was carried out at 42°C for 15min. A bulk PCR reaction mix was made consisting of RT² SYBR® Green qPCR Mastermix, H_2O and template cDNA, aliquots of which were then added to each well of the array plate. PCR was performed on an ABI Step-one instrument using a two-step Cycling Program of 95°C for 10min and 40 cycles of 95°C (15sec) and 60°C (60sec). Fold changes in expression of HLCs and fetal and adult hepatocytes over undifferentiated cells were calculated by the [illegible] method standardized against the combined expression of four housekeepers (*HPRT1*, *RPL13A*, *GAPDH* and *ACTB*) (*B2M* was unreliable in our experience as a housekeeping control). Data were analyzed using the RT² PCR Array Data software (SABiosciences). *P*-values were calculated for comparing groups based on a two-tailed Student’s t-test of the triplicate data for each gene. Of the 84 genes represented on the plates, 21 were excluded as inadequate either because detection in the fresh human adult hepatocytes was very low or because expression was more characteristic of other organs (e.g. CYP11B2 catalyzes the production of cortisol in the adrenal cortex). This left 63 enzymes by which to judge HLCs.

Proteomic analysis

Proteins identified by 2 or more peptides with $\geq$90% confidence, or by a single peptide with $\geq$99% confidence, were included in subsequent analyses. The dataset on whole cell extracts was filtered by statistical significance (P<0.05) using an unpaired t-test with Benjamini Hochberg adjustment. Proteins that were significantly upregulated >2-fold were interrogated against gene expression experiments deposited with the European Molecular Biology Lab / European Bioinformatics Institute (EMBL/EBI) Gene Expression Atlas (GEA; release 12.03.01; www.ebi.ac.uk/gxa/) [24 in main text] using the filter of up or down-regulated in at least one experiment on the GEA database.

Supplementary Figures

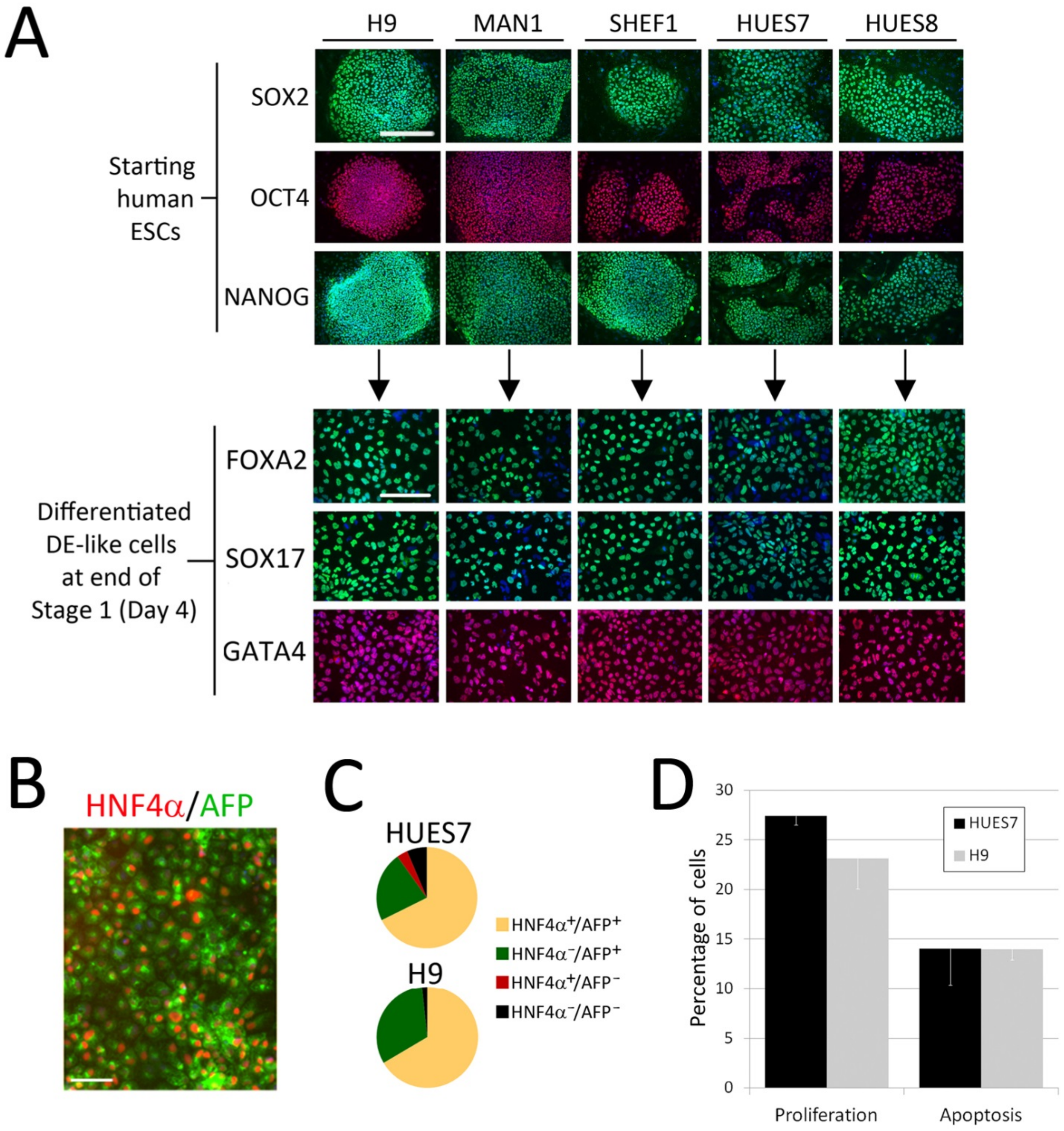

Supplementary Fig. 1. Characterization of the differentiation to definitive endoderm and hepatoblast-like cells. (A) Immunofluorescence for the differentiation of five human ESC lines to definitive endoderm-like cells indicative of those that are summarized graphically in Figure 2C. Size bars represent 150 µm (ESCs) and 50 µm (DE-like cells). (B) Immunofluorescence for HNF4α and AFP at the end of stage 2 (hepatoblast; example shown is H9). Size bar = 25 µm. (C) Pie charts of cell counting following immunocytochemistry at the end of Stage 2 from ≥ 3 differentiation experiments. (D) Proliferation and apoptosis in the HNF4α^{+}/AFP^{+} population assessed by BrdU incorporation and caspase 3 activity respectively. Mean +/- S.E. of three separate differentiation experiments shown.

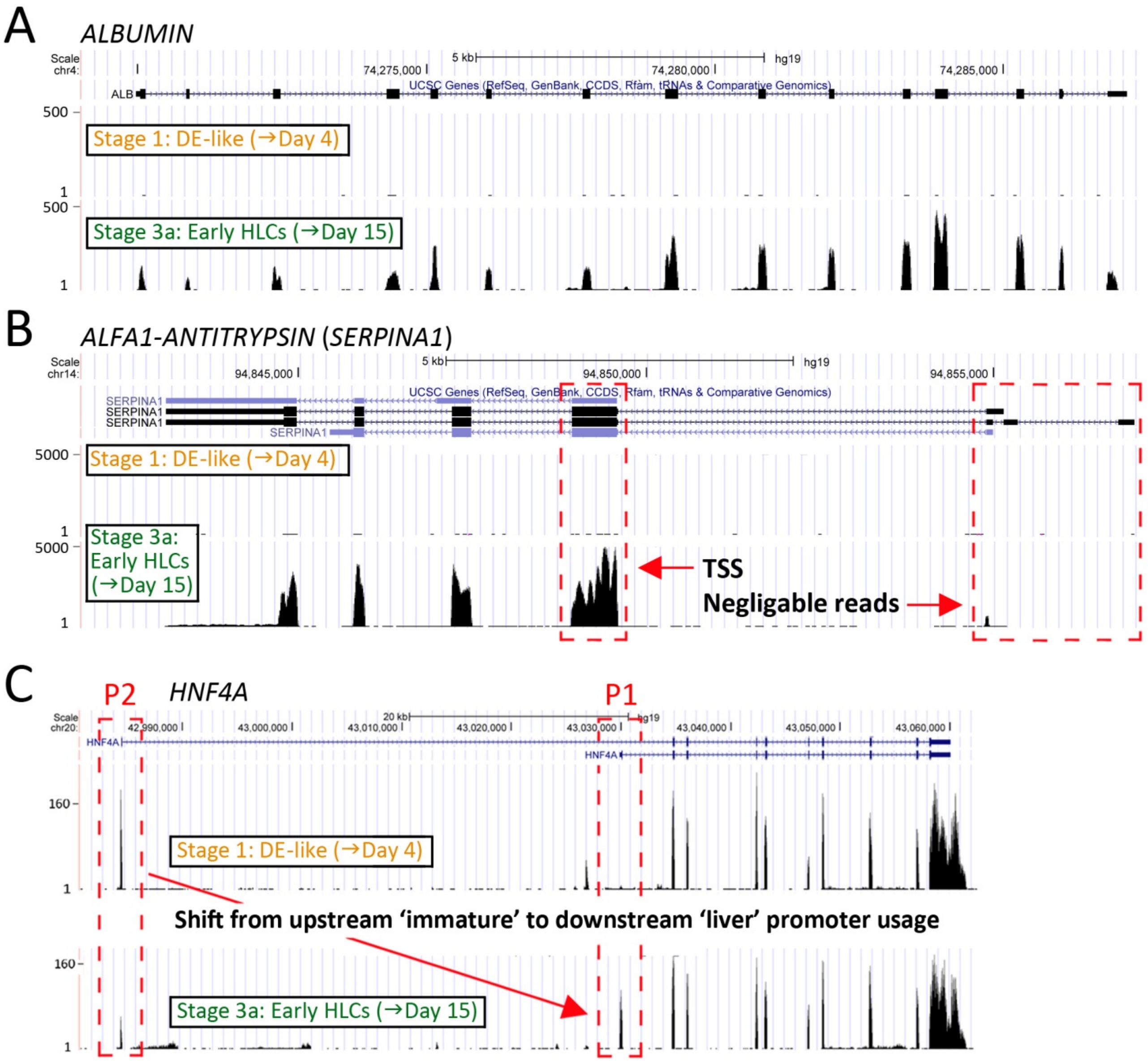

Supplementary Fig. 2. Additional characterization of hepatocyte-like cells. (A-C) RNA-seq read counts for albumin, alpha1-antitrypsin (*SERPINA1*) and *HNF4A* displayed on the y-axes and annotated using the UCSC Human Genome Browser for HUES7 HLCs. (A-B) Albumin and *AAT* expression was barely detected in DE-like cells but readily apparent at the end of Stage 3a. Red boxes in (B) show the transcription start site (TSS) at the first coding exon for AAT and negligible reads from upstream annotated exons (box to the right) concordant with data from human embryonic and adult liver. (C) Red boxes show the shift in usage from the P2 to the P1 promoter for *HNF4A* between DE-like cells and HLCs.

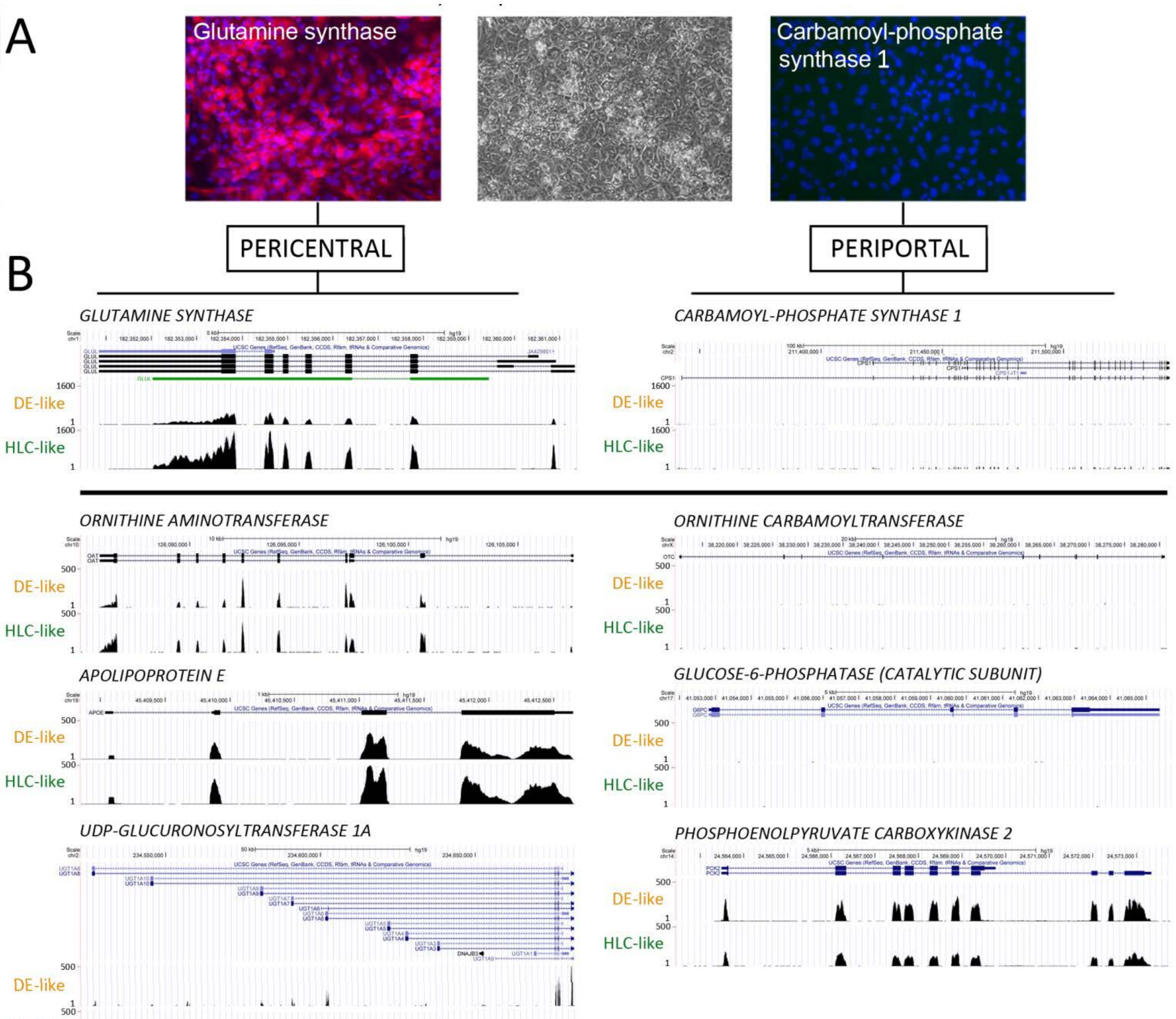

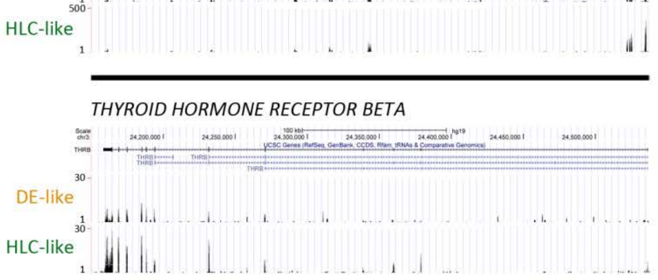

Supplementary Fig. 3. Hepatocyte-like cells show pericentral rather than periportal features. (A) Immunofluorescence of HUES7 HLCs showing detection of the pericentral hepatocyte marker, glutamine synthase, but not the periportal marker, carbamoyl-phosphate synthase 1. (B) Accompanying transcript analysis by RNA-seq for *glutamine synthase* and *carbmoyl-phosphate synthase 1* showing read counts on the same scale on the y-axis in both DE-like cells and HLCs. Further tracks are shown for other pericentral- and periportal-restricted transcripts [29]. Expression in HLCs of *glutamine synthase*, *ornithine aminotransferase*, *apolipoprotein E*, *UDP-glucuronosyltransferase 1A* and *thyroid hormone receptor β,* all indicative of pericentral hepatocytes [29], is greater than or equivalent to levels in DE-like cells. In contrast, periportal markers, *carbamoyl phosphate synthase 1*, *ornithine carbamoyltransferase* and the catalytic subunit of *glucose-6-phosphatase*, were not detected in DE-like cells or HLCs. *Phosphoenolpyruvate carboxykinase 2* was expressed in HLCs but at lower levels than those detected in DE-like cells.

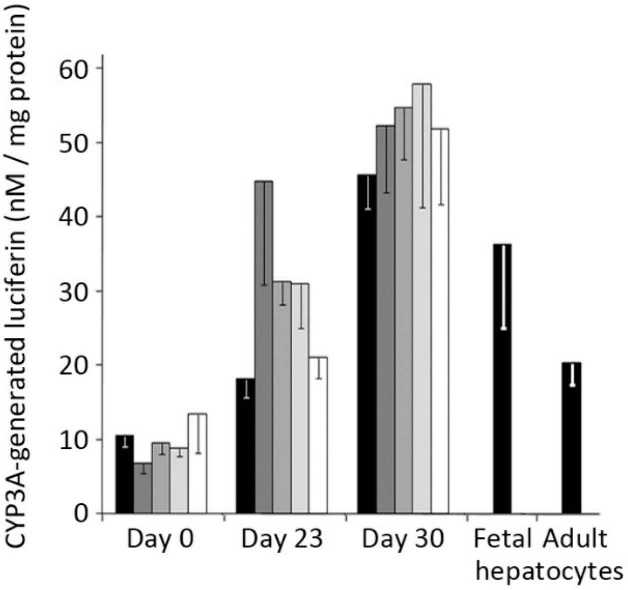

Supplementary Fig. 4. Luciferase assay for CYP3A activity is misleading for adult CYP3A4 activity. CYP3A luciferase assay at three points during differentiation compared to equivalent numbers of fetal and adult hepatocytes. Bar graphs show mean +/- S.E. from at least three independent experiments.

Supplementary Tables

Supplemental Table 1. Details of antibodies in immunofluorescence and immunoblotting.

Immunostaining details				
Primary Antibody				Secondary Antibody
Antigen	Dilution	Company	Fixative	Antibody
AAT	1:1000	Bethyl Lab Inc	4% PFA	donkey anti goat
AFP	1:100	R&D Systems	4% PFA	goat anti mouse IgG
Albumin	1:500	Sigma	4% PFA	donkey anti mouse IgG
CPS1	1:100	Abcam	Methanol	goat anti rabbit
CYP2A6	1:100	Origene	4% PFA	goat anti mouse IgG
FOXA2	1:150	R&D Systems	4% PFA	donkey anti goat
GATA4	1:500	Abcam	4% PFA	goat anti rabbit
GS	1:1000	BD Biosciences	4% PFA	goat anti mouse IgG
GSTp	1:500	Enzo	4% PFA	donkey anti rabbit IgG
HNF4α	1:500	Abcam	4% PFA	donkey anti rabbit IgG
HSP47	1:100	Abcam	4% PFA	goat anti mouse IgG
NANOG	1:20	R&D Systems	4% PFA	donkey anti goat
OCT4	1:150	Santa Cruz	4% PFA	donkey anti goat
SOX2	1:150	R&D Systems	4% PFA	goat anti mouse IgG
SOX17	1:150	R&D Systems	4% PFA	donkey anti goat

Immunoblotting details						
Primary Antibody			Blocking	Secondary Antibody		
Antigen	Dilution	Company	Agent	Antigen	Dilution	Company
α-fetoprotein	1:10000	Dako (A0008)	10% Milk	anti-rabbit	1:10000	GE Healthcare
β-actin	1:50000	Abcam (AB6276)	n/a	n/a (primary HRP conjugated antibody)		
CYP2A6	1:1000	Gift*	BSA	anti-chicken	1:10000	Invitrogen
CYP2D6	1:500	BD Gentest (458246)	10% Milk	anti-mouse	1:10000	Sigma
CYP3A	1:10000	BD Gentest	10% Milk	anti-mouse	1:10000	Sigma
CYPOR		Abcam (AB13513)				
FOXA2	1:1000	R&D Systems	10% Milk	anti-goat	1:5000	Dako
GATA4	1:1000	Abcam	10% Milk	anti-rabbit	1:10000	GE Healthcare
GSTp	1:5000	Enzo Life Sciences	10% Milk	anti-rabbit	1:10000	GE Healthcare
HSP47	1:1000	GE Healthcare	BSA	anti-rabbit	1:10000	GE Healthcare
SOX17	1:1000	R&D Systems	10% Milk	anti-rabbit	1:10000	GE Healthcare

Flow cytometry details

Primary Antibody			Secondary Antibody
**Antigen**	**Dilution**	**Company**	**Antibody**
AFP	1:50	R&D Systems	goat anti mouse
CYP2A6	1:100	Origene	goat anti mouse
GSTp	1:10	Enzo	goat anti rabbit
HSP47	1:200	Abcam	goat anti mouse

* Gift from R Juvonen, School of Pharmacy, Faculty of Health Sciences, University of Eastern Finland Abbreviations: α1-antitrypsin (AAT, officially designated SERPINA1), α-fetoprotein (AFP), carbamoyl phosphate synthase 1 (CPS1), cytochrome P450 (CYP) oxidoreductase (CYPOR), CYP2A6, CYP2D6, CYP3A isoforms, forkhead box factor A2 (FOXA2), GATA Binding Protein 4 (GATA4), glutamine synthetase (GS), glutathione S transferase π (GSTp), heat shock protein 47 (HSP47), hepatocyte nuclear factor 4α (HNF4α), sex-determining region Y box 2 (SOX2), SOX9 and SOX17.

Supplemental Table 2. Proteins upregulated in H9 hepatocyte-like cells.

Accession	Protein name	Fold increase	*p* value
Q96NY7	Chloride intracellular channel protein 6	9.58	0.002
P02489	Alpha-crystallin A chain	8.33	0.027
P14174	Macrophage migration inhibitory factor	3.60	0.001
P12277	Creatine kinase B-type	3.50	0.000
P07585	Decorin	3.41	0.004
P51884	Lumican	3.30	0.004
P04792	Heat shock protein beta-1	3.28	0.003
P35670	Copper-transporting ATPase 2	3.20	0.039
P40261	Nicotinamide N-methyltransferase	3.13	0.003
Q9HCY8	Protein S100-A14	3.05	0.009
P02671	Fibrinogen alpha chain	2.91	0.012
P08123	Collagen alpha-2(I) chain	2.88	0.004
P21266	Glutathione S-transferase Mu 3	2.86	0.004
P43320	Beta-crystallin B2	2.81	0.012
Q9UK22	F-box only protein 2	2.80	0.004
P09525	Annexin A4	2.79	0.007
P00352	Retinal dehydrogenase 1	2.69	0.014
P84157	Matrix-remodeling-associated protein 7	2.61	0.006
P27487	Dipeptidyl peptidase 4	2.59	0.004
Q969E4	Transcription elongation factor A protein-like 3	2.53	0.004
P02458	Collagen alpha-1(II) chain	2.52	0.012
P13473	Lysosome-associated membrane glycoprotein 2	2.52	0.000
P07686	Beta-hexosaminidase subunit beta	2.51	0.003
P29966	Myristoylated alanine-rich C-kinase substrate	2.50	0.005
P05787	Keratin, type II cytoskeletal 8	2.48	0.008
Q99836	Myeloid differentiation primary response protein MyD88	2.48	0.014
P10619	Lysosomal protective protein	2.40	0.002
P02538	Keratin, type II cytoskeletal 6A	2.39	0.001
P01009	Alpha-1-antitrypsin	2.36	0.016
P02751	Fibronectin	2.35	0.001
Q15582	Transforming growth factor-beta-induced protein ig-h3	2.34	0.002
P07858	Cathepsin B	2.33	0.002
P15586	N-acetylglucosamine-6-sulfatase	2.32	0.003
P21980	Protein-glutamine gamma-glutamyltransferase 2	2.31	0.001
P80303	Nucleobindin-2	2.30	0.002
P80723	Brain acid soluble protein 1	2.29	0.001
P02771	Alpha-fetoprotein	2.28	0.012
P02679	Fibrinogen gamma chain	2.27	0.016
P12111	Collagen alpha-3(VI) chain	2.26	0.005
P42356	Phosphatidylinositol 4-kinase alpha	2.23	0.007
P09455	Retinol-binding protein 1	2.23	0.002
Q92820	Gamma-glutamyl hydrolase	2.22	0.001
P09211	Glutathione S-transferase P	2.21	0.001

P35270	Sepiapterin reductase	2.21	0.004
P34059	N-acetylgalactosamine-6-sulfatase	2.20	0.004
P08473	Neprilysin	2.18	0.016
Q969L2	Protein MAL2	2.18	0.010
O60443	Non-syndromic hearing impairment protein 5	2.18	0.014
P27216	Annexin A13	2.15	0.010
P13647	Keratin, type II cytoskeletal 5	2.15	0.023
P48735	Isocitrate dehydrogenase [NADP], mitochondrial	2.12	0.002
P02675	Fibrinogen beta chain	2.12	0.012
P05783	Keratin, type I cytoskeletal 18	2.09	0.014
P51649	Succinate-semialdehyde dehydrogenase, mitochondrial	2.08	0.006
P05161	Ubiquitin-like protein ISG15	2.07	0.008
O14773	Tripeptidyl-peptidase 1	2.07	0.005
Q96IU4	Abhydrolase domain-containing protein 14B	2.04	0.010
P30085	UMP-CMP kinase	2.04	0.006
P52565	Rho GDP-dissociation inhibitor 1	2.03	0.000
P16278	Beta-galactosidase	2.02	0.001
O95865	N(G),N(G)-dimethylarginine dimethylaminohydrolase 2	2.02	0.002
P54725	UV excision repair protein RAD23 homolog A	2.02	0.017
P08729	Keratin, type II cytoskeletal 7	2.01	0.001

Table 2 shows the list of proteins where the mean triplicate value was increased more than 2-fold and statistically significant compared to undifferentiated cells.

Supplementary References

1. Trapnell C, Salzberg SL. How to map billions of short reads onto genomes. Nat Biotechnol 2009;27:455-457.
2. Kent WJ, Sugnet CW, Furey TS, Roskin KM, Pringle TH, Zahler AM, Haussler D. The human genome browser at UCSC. Genome Res 2002;12:996-1006.
